# The Coming Meeting of the American Dental Association

**Published:** 1886-04

**Authors:** 


THE COMING MEETING OF THE AMERICAN DENTAL ASSOCIATION.--Although this Association voted to select San Francisco as its place of meeting for 1886, yet it has finally been determined to once more return to Niagara Falls. No doubt our California brethren will be greatly disappointed at this change, for we learn that preparations had already been made to welcome the members of the Association in the Golden Gate City. Judging from an experience in 1882, the dental profession in attendance would have been most hospitably entertained by the California dentists and the trip would have been a delightful one for all who have never visited the Pacific Coast. We think a great opportunity as well as pleasure has been lost by this change.

